# Unsupervised Clustering of Routine Inflammatory Markers in Cardiogenic Shock Reveals Phenotypic Heterogeneity Without Prognostic Utility

**DOI:** 10.3390/jpm16020096

**Published:** 2026-02-06

**Authors:** Song Peng Ang, Jackson Rajendran, Yashika Gupta, Jia Ee Chia, Shana John, Madison Laezzo, Chukwudi Ikeano, Eunseuk Lee, Jose Iglesias

**Affiliations:** 1 Division of Cardiology, University of Arizona, Tucson, AZ 85724, USA; songpengang@arizona.edu; 2 Department of Medicine, Rutgers Health Community Medical Center, Toms River, NJ 08755, USA; jack.gsjc@hotmail.com (J.R.); chukwudiikeano92@gmail.com (C.I.); esesok@gmail.com (E.L.); 3 Department of Medicine, Gadag Institute of Medical Sciences, Gadag 582103, India; yashika.gupta022@gmail.com; 4 Department of Medicine, Texas Tech University Health Science Center, El Paso, TX 79905, USA; jiaee1223@gmail.com; 5 Department of Family Medicine, Hackensack Meridian Ocean Medical Center, Brick, NJ 08724, USA; shana.hannah.john@gmail.com; 6 Department of Medicine, Hackensack Meridian School of Medicine, Nutley, NJ 07110, USA; madison.laezzo@hmhn.org

**Keywords:** cardiogenic shock, inflammation, neutrophil-to-lymphocyte ratio, phenotyping, critical care

## Abstract

**Background**: Cardiogenic shock is a heterogeneous syndrome in which systemic inflammation may contribute to cardiovascular risk and adverse outcomes beyond hemodynamic compromise alone. **Methods**: We conducted a retrospective multicenter cohort study using the eICU Collaborative Research Database (2014–2015) to identify inflammatory phenotypes among adults admitted to intensive care units with cardiogenic shock. Inflammatory indices derived from admission hematologic parameters (including NLR, PLR, MLR, NPAR, SII, SIRI, and AISI) were analyzed using principal component analysis, followed by hierarchical and k-means clustering to identify biologically distinct inflammatory phenotypes. Clinical characteristics and short-term outcomes were compared across clusters. **Results**: Among 419 patients, two phenotypes were identified. Cluster 1 (n = 52) was characterized by older age, a higher prevalence of chronic kidney disease (CKD), more advanced renal and hepatic dysfunction, along with a hyperinflammatory, lymphopenic profile. Cluster 2 (n = 367) exhibited comparatively lower inflammatory indices and less biochemical derangement. There was a significant difference in the prevalence of CKD, the need for mechanical ventilation, and history of malignancy between clusters. Despite clear biological separation, short-term clinical outcomes, including rates of acute kidney injury requiring renal replacement therapy, vasopressor use, hospital length of stay, and in-hospital mortality, were similar across clusters. **Conclusions**: These findings suggest that cardiogenic shock encompasses distinct inflammatory phenotypes, but inflammatory clustering based on routine admission laboratory data alone may have limited utility for short-term risk stratification.

## 1. Introduction

Cardiogenic shock (CS) is distinguished by inadequate cardiac output, impaired tissue perfusion, and multiorgan dysfunction [1,2]. While acute myocardial infarction (MI) remains a leading cause of CS, non-ischemic etiologies, including worsening heart failure, valvular heart disease, and inflammatory cardiomyopathies, are being increasingly recognized. Despite advances in revascularization, critical care, and mechanical circulatory support, the mortality and morbidity associated with CS remain high [3,4,5]. CS represents a heterogeneous syndrome with distinct hemodynamic and biological profiles beyond a purely ischemic mechanism. Mounting evidence suggests that systemic inflammation and immune dysfunction play a crucial role in CS pathophysiology and clinical outcomes.

Peripheral blood-derived inflammatory ratios, including the neutrophil-to-lymphocyte ratio (NLR), platelet-to-lymphocyte ratio (PLR), and monocyte-to-lymphocyte ratio (MLR), systemic immune-inflammation index (SII), systemic inflammatory response index (SIRI), aggregate index of systemic inflammation (AISI), Platelet-Lymphocyte Ratio (PLR) and Monocyte-Lymphocyte Ratio (MLR) have been proposed as readily available markers of inflammatory activity [6,7,8]. In CS, these indices correlate with disease severity and outcomes, indicating an initial hyperinflammatory phase followed by immune exhaustion [9]. Elevated SIRI and SII values have been independently associated with higher long-term mortality in chronic heart failure [10,11].

Unsupervised clustering has recently been used to define clinically relevant phenotypes in sepsis, heart failure, and acute coronary syndromes. Recent biomarker-based analysis of cardiogenic shock has identified distinct subphenotypes with differing inflammatory profiles, organ dysfunction patterns, and mortality risk, demonstrating that data-driven clustering can yield prognostically meaningful CS endotypes [12,13,14].

While most existing CS and MI-CS studies have evaluated these indices individually or in limited combinations, to our knowledge, no study has systematically applied PCA followed by cluster analysis to a comprehensive panel of hematologic inflammation indices (NLR, PLR, MLR, SII, SIRI, AISI) within a cardiogenic shock cohort to identify distinct phenotypic subgroups. In this study, we analyze a consecutive cohort of patients with cardiogenic shock, quantify these inflammatory indices upon admission (SII, AISI, NLR, PLR, MLR, SIRI), and employ Principal Component Analysis (PCA) and clustering to identify distinct inflammatory phenotypes within the CS population. Thus, our study differs from prior SCAI-based staging and transcriptomic or cytokine-derived clustering by focusing on unsupervised phenotyping derived solely from routinely available hematologic indices at the time of ICU admission.

## 2. Methods

**Figure 1:**
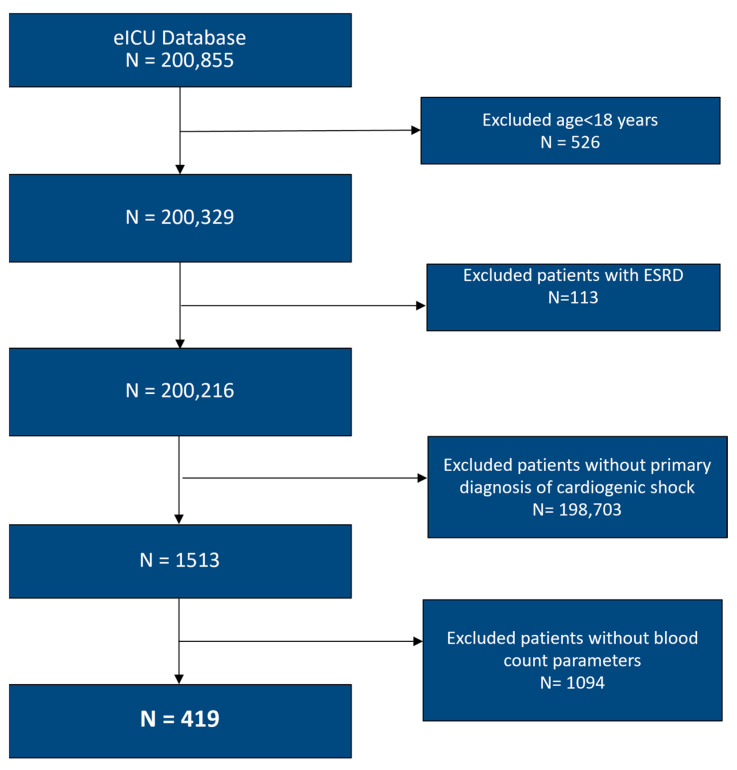
Flowchart of selection of participants. ESRD: End-Stage Renal Disease.

We conducted a retrospective, multicenter cohort study using the eICU Collaborative Research Database, which contains de-identified data from more than 200 intensive care units (ICUs) across the United States, collected between 2014 and 2015 [15]. Adult patients admitted to an ICU with cardiogenic shock were identified using International Classification of Diseases, 9th Revision, Clinical Modification (ICD-9-CM) codes. In constructing the cohort, we excluded patients with pre-existing end-stage kidney disease, as well as those with missing laboratory variables required to calculate the inflammatory indices used in the analysis. Other inflammatory biomarkers, such as ESR, CRP, and ferritin, were recorded sporadically in the database and were therefore not included in the PCA or clustering analysis. Details of cohort assembly and exclusions are shown in Figure 1.

The study is exempt from institutional Clinical Modification due to its retrospective design, lack of direct patient management, and the security schema, for which the re-identification risk was certified as complying with safe harbor standards by an independent privacy expert (Privacert, Cambridge, MA, USA) (Health Insurance Portability and Accountability Act Certification No. 1031219-2) [15]. Informed Consent was waived due to the retrospective nature of the study and the deidentified nature of the dataset.

Demographic variables, comorbidities, intervention, and initial laboratory parameters obtained on admission were extracted from electronic health records during the ICU hospitalization. Inflammatory markers, NLR, PLR, MLR, NPAR, SII, SIRI, and AISI were calculated using standard formulas as follows: NLR = Neutrophil count/Lymphocyte count; PLR = Platelet count/Lymphocyte count; MLR = Monocyte Count/Lymphocyte Count; SII = (Platelet count × Neutrophil count/Lymphocyte count; NPAR = (Neutrophil percentage of total white blood cell count (%) × 100)/Albumin (g/dL); SIRI = (Neutrophil count × Monocyte count)/Lymphocyte count; AISI = (Neutrophil count × Platelet count × Monocyte count)/Lymphocyte count. AKI was defined based on the 2012 Kidney Disease: Improving Global Outcomes (KDIGO) criteria.

### 2.1. Outcomes

The primary objective was to determine whether there were differences in phenotypes among patients with cardiogenic shock using inflammatory markers from routine laboratory testing obtained on admission. Secondary outcomes included complications such as mechanical ventilation, AKI requiring renal replacement therapy (RRT), vasopressor requirement, hospital length of stay, and hospital mortality.

### 2.2. Statistical Analysis

Variables were expressed as mean and standard deviation for normally distributed variables, and as median and interquartile range for data that were not normally distributed. The Shapiro–Wilk and Kolmogorov–Smirnov tests were used to determine normality. Clinical and laboratory variables were compared between clusters using the chi-square test for categorical variables and the Mann–Whitney U test for continuous variables.

To determine latent phenotypes based on admission hematologic inflammation indices, without using outcome information, we performed an unsupervised analysis consisting of PCA followed by hierarchical clustering and k-means clustering. PCA was performed on standardized admission values; the number of principal components retained was guided by the scree plot breakplot (elbow). The Kaiser–Meyer–Olkin (KMO) test and Bartlett’s test of Sphericity were used to assess the suitability for PCA. The representative variables for the principal components were selected based on factor loadings. Following dimensionality reduction, hierarchical clustering was conducted using squared Euclidean distance with Ward’s linkage method. The number of clusters was determined by inspecting the dendrogram and agglomeration schedule, considering changes in linkage distance and interpretability. K-means clustering was then performed using the selected number of clusters; cluster assignment was based on the cluster whose centroid minimized the squared Euclidean distance. Final cluster assignments were compared using ANOVA. All analyses were conducted using IBM SPSS Statistics version 27 (Chicago, IL, USA) with the significance threshold set at *p* < 0.05.

As the inflammatory indices are calculated from overlapping leukocyte and platelet count indices, which results in mathematical coupling and collinearity, we assessed the relationships among the inflammatory indices used in the PCA. A Spearman’s rank correlation matrix was constructed, including SII, AISI, NLR, PLR, MLR, SIRI, and NPAR. Using the entire analytic cohort (419 cases), correlations were estimated and considered statistically significant if 2-sided *p*-values were <0.05. The resulting correlation matrix is reported in Supplementary Table S1, which supports the use of PCA before cluster analysis to reduce collinearity.

To further explore the structure and redundancy of these inflammatory indices, we then performed an additional sensitivity analysis, employing a separate PCA restricted to NLR, PLR, MLR, and NPAR. We then applied *k-means* clustering with regression-based factor scores from the two-component PCA of NLR, PLR, MLR, and NPAR as entry variables, using the same number of clusters as in the primary analysis. We then compared the primary and key secondary outcomes between these PCA-derived inflammatory clusters (Supplemental Tables S2–S4).

## 3. Results

Among 419 ICU patients admitted with cardiogenic shock, the median age was 67 years (interquartile range [IQR]: 58–77), the mean body mass index was 27.6 kg/m^2^ (IQR: 24.0–32.5), 252 (60.1%) were male, and 321 (76.6%) were Caucasian.

Sampling adequacy for principal component analysis was acceptable (Kaiser–Meyer–Olkin statistic 0.57), and Bartlett’s test of sphericity was highly significant (χ^2^ = 2863, df = 21, *p* < 0.001), confirming that the correlation structure among the inflammatory indices was suitable for dimension reduction. Communalities after extraction were high for SII, AISI, NLR, MLR, and SIRI (0.80–0.91), and moderate for PLR (0.49) and NPAR (0.33), indicating that most of the variance in these measures was captured by the common components (Table 1). The first principal component explained 60.7% of the total variance, and the second explained an additional 13.8%, for a cumulative 74.5%; these two components were therefore retained as inputs for the clustering analysis.

**Figure 2:**
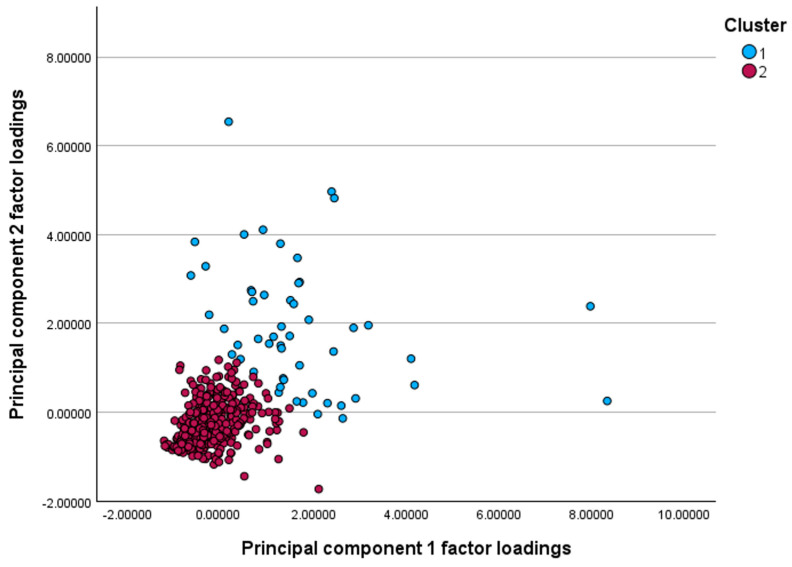
Scatterplot of principal component factor loadings illustrating two distinct clusters. Cluster 1 (blue) shows generally higher loadings on both components, whereas Cluster 2 (red) demonstrates lower loading scores, indicating separation of patients into two inflammatory profiles.

Based on hierarchical clustering of PCA factor scores, a two-cluster solution (Cluster 1, n = 52; Cluster 2, n = 367) was retained as higher-order solutions yielded very small, clinically uninformative groups (Figure 2 and Figure S1). Differences in mean factor scores between clusters were highly significant for both Factor 1 (F = 273, ANOVA *p* < 0.0001) and Factor 2 (F = 471, ANOVA *p* < 0.0001), indicating good separation.

**Figure 3:**
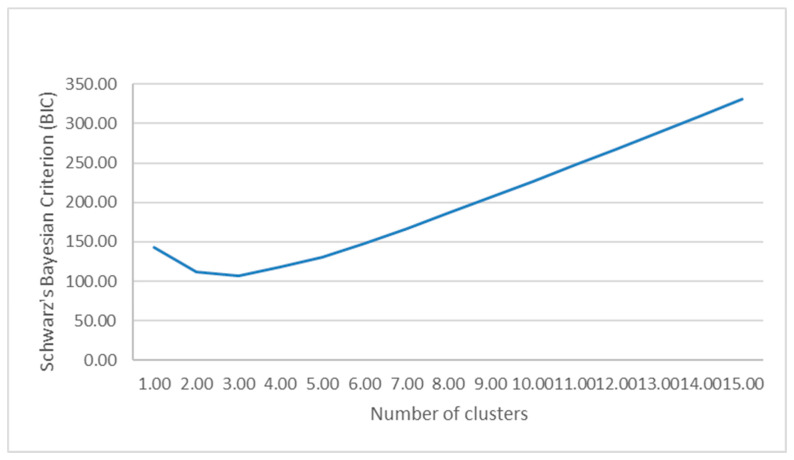
Graph demonstrating the association between cluster number and Schwartz’s Bayesian Information Criterion (BIC). Of note, the model fit is optimized when transitioning from a one-cluster solution to a two-cluster solution with only minimal changes for a higher number of cluster solutions.

A plot of the Bayesian Information Criterion (BIC) across candidate models showed the largest improvement in fit when moving from a one- to a two-cluster solution, with only minor additional changes for higher numbers of clusters (Figure 3). On this basis, the 2-cluster solution was retained for all subsequent analyses. In addition, both the maximum BIC change and the ratio of change reached their peak with the two-cluster solution, indicating that this is where the most significant increase in model fit occurs.

Patients in Cluster 1 were older (median 72 vs. 67 years, *p* = 0.01), more often male (75% vs. 58%, *p* = 0.02), and more likely to have chronic kidney disease (38.5% vs. 19.4%, *p* = 0.002) and malignancies than those in Cluster 2. The prevalence of diabetes mellitus, cardiomyopathy, cerebrovascular disease, and chronic obstructive pulmonary disease was similar between clusters, and the ethnic distribution was comparable, with the Caucasian race predominating in both groups (Table 2 and Table 3).

Laboratory values at ICU admission demonstrated an overall pattern of greater physiologic derangement in Cluster 1, characterized by more pronounced renal dysfunction and broader metabolic disturbance. Blood urea nitrogen (median 49 vs. 33 mg/dL, *p* < 0.001) and serum creatinine (2.4 vs. 1.6 mg/dL, *p* = 0.02) were higher, and cluster 1 also had lower serum sodium (134 vs. 137 mmol/L, *p* < 0.001) and chloride (95 vs. 102 mmol/L, *p* = 0.01), slightly higher potassium (4.6 vs. 4.3 mmol/L, *p* = 0.05), and higher total bilirubin (1.5 vs. 0.95 mg/dL, *p* = 0.03). Lactic acid levels were lower in Cluster 1 (2.2 vs. 3.5 mmol/L, *p* = 0.03).

Across complete blood count-derived inflammatory indices, Cluster 1 displayed a more inflammatory and lymphopenic phenotype with higher neutrophils (9.8 vs. 7.5 × 10^3^/µL, *p* < 0.001), markedly lower lymphocytes (0.51 vs. 1.57 × 10^3^/µL, *p* < 0.001), and a substantially increased NLR (20.6 vs. 4.8, *p* < 0.001). Composite indices incorporating neutrophils, platelets, monocytes, and lymphocytes were similarly elevated in Cluster 1: SII (4869 vs. 980, *p* < 0.001), PLR (460.9 vs. 129.1, *p* < 0.001), MLR (16.3 vs. 4.4, *p* < 0.001), SIRI (164.0 vs. 33.4, *p* < 0.001), and NPAR (0.27 vs. 0.22, *p* < 0.001). Albumin (3.1 vs. 3.4 g/dL, *p* = 0.003) and hemoglobin (11.5 vs. 12.4 g/dL, *p* = 0.01) were lower in Cluster 1, consistent with a more catabolic and inflamed state. In contrast, AISI was lower in Cluster 1 than in Cluster 2 (3.7 vs. 6.8 × 10^9^/L, *p* < 0.001), which is consistent with AISI being more sensitive to differences in platelet and monocyte components (and their interaction) rather than tracking lymphopenia-driven separation captured by NLR, SII, and SIRI.

Despite these marked differences in inflammatory burden and organ dysfunction at presentation, subsequent clinical outcomes were broadly similar between clusters. The incidence of acute kidney injury was high in both groups (67.3% vs. 64.9%, *p* = 0.73), as was the need for renal replacement therapy (13.5% vs. 13.4%, *p* = 0.98). Rates of acute myocardial infarction (25.0% vs. 32.2%, *p* = 0.29) and other significant comorbidities were comparable. Percutaneous coronary intervention was numerically more frequent in Cluster 2 (1.9% vs. 10.4%, *p* = 0.05).

With respect to resource use and mortality, mechanical ventilation was more common in Cluster 2 (51.9% vs. 69.7%, *p* = 0.01), whereas vasopressor use was frequent and similar in both groups (76.9% vs. 81.7%, *p* = 0.41). Overall hospital mortality was 34.8% and did not differ significantly between Cluster 1 and Cluster 2 (40.4% vs. 34.1%, *p* = 0.37). Early mortality within 24 h of admission (13.5% vs. 19.4%, *p* = 0.30) and median hospital length of stay (4.0 vs. 4.1 days, *p* = 0.86) were similarly comparable. Together, these findings suggest that a smaller subset of cardiogenic shock patients presents with a more inflammatory, renal dysfunction phenotype. Still, this pattern was not associated with detectable differences in short-term mortality or length of stay in this cohort.

As anticipated, there were moderate to robust positive correlations across the correlation matrix (Supplementary Table S1). Correlation coefficients across inflammatory indices SII, AISI, NLR, PLR, and SIRI ranged from 0.72 to 0.91 and were statistically significant (all *p* values < 0.001). These findings indicate substantial shared variance in the information they capture. NPAR correlated with the other indices to a lesser extent, with correlation coefficients ranging from 0.38 to 0.91. This evidence supports the idea that due to mathematical coupling, these inflammatory indices are highly interrelated.

The sensitivity analysis was performed using NLR, PLR, MLR, and NPAR using a separate PCA. The two-factor solution accounted for 75.6% of the total variance (KMO = 0.65; Bartlett’s test, *p* < 0.001). The first component was characterized by higher PLR and MLR, with contributions from NLR, whereas the second component was driven primarily by NPAR, with additional loading from NLR (Supplementary Table S2).

Based on the PCA of abbreviated inflammatory components, K-means clustering yielded 2 groups comprising 320 (76.4%) and 99 (23.6%) patients, respectively. The larger group had mean factor scores below zero on both components, consistent with a lower inflammatory burden. In contrast, the smaller group had mean scores more than one standard deviation above 0 on both components, indicating a higher inflammatory burden (Supplementary Table S3). Despite this clear separation in component space, in-hospital mortality did not differ between the PCA-derived clusters. The higher inflammation cluster had more dialysis-dependent acute kidney injury and, somewhat unexpectedly, lower rates of invasive mechanical ventilation; however, these differences did not alter the overall finding that admission inflammatory phenotypes provided limited discrimination ability for short-term mortality in this cohort (Supplementary Table S4).

**Figure 4:**
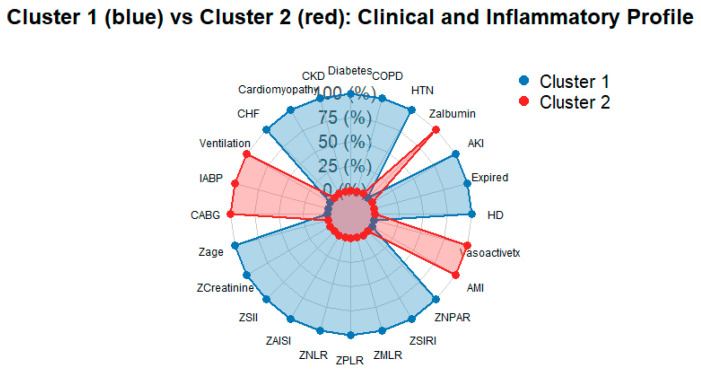
Spider Plot showing standardized baseline features of the two clusters. Axes include inflammatory indices, age, kidney function, and selected comorbidities and support measures. Higher values indicate measurements above the cohort mean, highlighting the more inflamed, sicker profile of cluster 1 compared with cluster 2.

**Figure 5:**
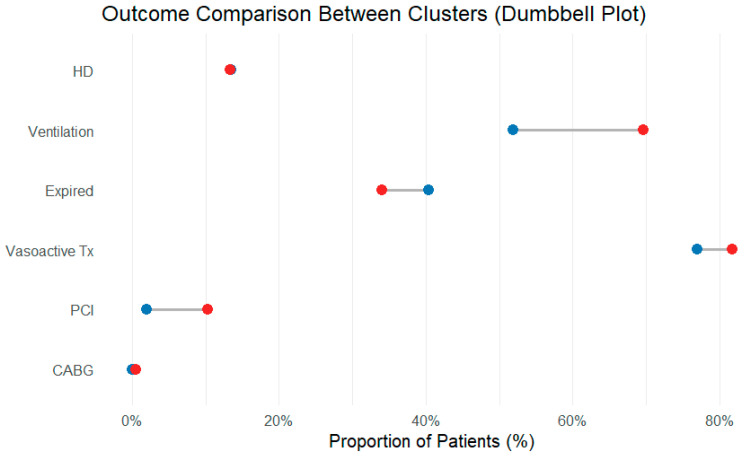
Dumbbell plot comparing the proportions of major outcomes in each cluster. Each line connects the cluster-specific proportions for a given endpoint; the horizontal distance between the points represents the absolute difference. The short distance for most outcomes illustrates that, despite different inflammatory profiles, the short-term clinical results were similar. Blue dots: cluster 1; red dots: cluster 2.

To summarize the overall pattern, a spider plot was constructed, illustrating that cluster 1 has higher standardized inflammatory indices, older age, and worse renal function than cluster 2, despite similar uses of organ support and outcomes (Figure 4). The accompanying dumbbell plot shows only modest absolute differences in the proportions of primary and secondary clinical endpoints between clusters, underscoring that marked inflammatory and biochemical separation and admission did not translate into large differences in short-term clinical outcomes (Figure 5).

## 4. Discussion

In this multicenter cohort of critically ill patients with cardiogenic shock, we identified two distinct inflammatory phenotypes using principal component analysis followed by hierarchical and k-means clustering of routinely available hematologic indices. One cluster was characterized by higher age, a greater burden of chronic kidney disease, more profound renal and hepatic dysfunction, and a strikingly hyperinflammatory, lymphopenic profile. The other cluster showed comparatively lower inflammatory indices and less severe biochemical derangement. Despite these marked biological differences at presentation, short-term outcomes, including need for renal replacement therapy, length of stay, and in-hospital mortality, were broadly similar between clusters.

An important contextual consideration is that the current database captures a highly selected population of patients with advanced cardiogenic shock requiring ICU-level care. By the time patients enter this cohort, most have already developed severe hemodynamic compromise and multi-organ dysfunction, and many have undergone time-sensitive interventions. At this late stage of illness, the dominant drivers of outcome are likely to be global hemodynamics, cumulative organ injury, and treatment factors, which may overshadow any incremental prognostic contribution of baseline inflammatory markers. This selection of uniformly high-risk patients provides a plausible explanation for why biologically distinct inflammatory phenotypes did not translate into clear differences in short-term mortality or other outcomes in our analysis.

These findings both align with and diverge from prior work on inflammation in cardiogenic shock [16]. Existing studies have consistently shown that higher NLR, SII, SIRI, and related indices track with greater illness severity and worse outcomes in acute coronary syndromes and chronic heart failure, and that inflammatory markers vary across SCAI shock stages [17,18,19]. In that context, the hyperinflammatory, lymphopenic phenotype we observed in Cluster 1 is biologically plausible: the higher neutrophil counts, profound lymphopenia, elevated SII and SIRI, and lower albumin all point toward an activated innate immune response coupled with impaired adaptive immunity and a catabolic milieu. This pattern parallels immune signatures described in septic and mixed-shock states, and supports the concept that cardiogenic shock is not purely a hemodynamic syndrome, but rather one with substantial immunologic heterogeneity [16,20]. The unexpectedly lower lactate levels and higher use of mechanical ventilation in the less inflamed cluster likely reflect differences in illness severity, timing of blood sampling, and resuscitation rather than a direct relationship with the inflammatory profile itself.

However, the absence of a clear mortality gradient between clusters contrasts with the more straightforward risk gradients reported when inflammatory markers are examined individually or when shock severity is staged using clinical variables. Several factors may contribute. First, our cohort represents a highly selected, uniformly high-risk population in the ICU. Once patients reach an advanced shock state requiring intensive care, the incremental prognostic contribution of baseline inflammatory indices may be attenuated by the dominant effects of macro-hemodynamics, end-organ injury, and the availability and timing of advanced therapies such as revascularization and mechanical circulatory support [21]. In other words, by the time patients enter this dataset, they may already be on the “plateau” of a high-risk curve where additional biological separation does not translate into further measurable differences in short-term mortality.

Second, the temporal dimension of the immune response is not captured here. We relied on a single set of laboratory values at or near ICU admission. Experimental and clinical data suggest that cardiogenic shock may follow a biphasic immune trajectory, with an early hyperinflammatory phase followed by immune exhaustion and susceptibility to secondary insults [22]. A single snapshot may conflate patients at different points along this trajectory; some “hyperinflammatory” patients may be on the upslope of a potentially recoverable response, whereas others in the “less inflamed” cluster may already be entering a hypo-inflammatory or immunoparalyzed phase. Without serial measurements, we cannot determine whether cluster membership is stable over time, nor whether transitions between phenotypes carry prognostic significance. This is a key distinction from sepsis “endotypes” based on longitudinal transcriptomic or cytokine data.

Third, the clusters were derived entirely from inflammatory indices and did not incorporate granular hemodynamic, etiologic, or treatment variables. The eICU database does not reliably capture Society of Angiography and Interventions (SCAI) shock staging, timing of coronary angiography, completeness of revascularization, escalation to intra-aortic balloon pump or temporary mechanical circulatory support, or standardized use of guideline-directed medical therapy. These factors are strong determinants of survival in cardiogenic shock and may obscure or override the prognostic effect of the inflammatory phenotype. It is plausible that the hyperinflammatory cluster would show a differential response to therapies such as early revascularization, more aggressive decongestion, or future immunomodulatory strategies, but such effect modification cannot be evaluated in this dataset.

The biological features of the clusters also warrant careful interpretation. The hyperinflammatory cluster had higher BUN and creatinine, lower sodium and chloride, and higher bilirubin, consistent with more severe renal and hepatic congestion or hypoperfusion. At the same time, lactic acid levels were lower in this group. This apparent paradox may reflect timing of measurement (for example, sampling after initial resuscitation), differences in vasopressor exposure, or a phenotype in which microcirculatory and metabolic dysfunction are less prominent than venous congestion and organ edema. Alternatively, it may suggest that inflammatory dysregulation and venous congestion can proceed somewhat independently of global tissue hypoxia in certain cardiogenic shock trajectories. This highlights the danger of equating “inflammation” with “severity” in a linear fashion; immune activation may be partially uncoupled from traditional hemodynamic surrogates [23].

Clinically, our data suggest that inflammatory indices can identify a biologically distinct subgroup of cardiogenic shock patients, but do not support using these clusters for risk stratification or treatment allocation in isolation [24]. The hyperinflammatory phenotype may still have value as a biomarker of immune activation and comorbidity burden, particularly renal dysfunction, and could be used to enrich future interventional trials testing immunomodulatory or organ-support strategies. More broadly, the analytic framework combining PCA with clustering of routinely available laboratory indices illustrates a scalable approach that could be applied to other ICU populations and data sources to derive reproducible phenotypes [20]. Yet any move toward phenotype-guided therapy will require prospective validation, integration with hemodynamic and imaging data, and evaluation of treatment–phenotype interactions [25].

Several limitations should temper the interpretation of the results. Although our clustering approach identified two clearly distinct inflammatory profiles, the lack of separation in short-term outcomes suggests that, in the late ICU stage of cardiogenic shock, these phenotypes should, at this point in time, be interpreted as descriptive biological states rather than actionable clinical subgroups. The hyper-inflammatory, lymphopenic cluster appears to capture a pattern of immune activation, renal dysfunction, and hepatic congestion, but our data do not support using these clusters alone to guide immediate risk stratification or treatment decisions. Importantly, the absence of an association with in-hospital mortality does not exclude potential relevance for longer-term outcomes, complications, or differential response to therapies, particularly if inflammatory phenotypes were integrated with hemodynamic, etiologic, and therapeutic data or assessed longitudinally over the course of shock. Prospective studies incorporating serial inflammatory measurements and testing for treatment-phenotype interactions will be needed to determine whether these biologically defined groups have prognostic or predictive utility.

Several methodological limitations should be acknowledged. This is a retrospective analysis of a multicenter, database drawn from U.S. ICUs participating in a tele-ICU network [15]. Selection into the eICU program and local practice patterns may limit the generalizability of findings to centers with different case mixes, resource availability, or shock management strategies. Misclassification of cardiogenic shock diagnosis is possible, as coding and registry fields may not fully distinguish primary from mixed shock states or capture the underlying etiology (acute myocardial infarction versus decompensated chronic cardiomyopathy, valvular disease, myocarditis, or post-cardiotomy shock). Residual and unmeasured confounding are therefore likely. One cluster was substantially smaller than the other (52 vs. 367 patients), which may limit the statistical power to detect modest differences in outcomes and increase the risk that some findings for the smaller group are unstable. This imbalance also reduces generalizability, as the hyper-inflammatory phenotype represents a minority of ICU cardiogenic shock cases in this dataset and may not be sampled similarly in other cohorts.

As the eICU database relies on ICD-9 coding and lacks reliable data on SCAI stage, shock etiology, timing, completeness of revascularization, and temporary mechanical circulatory support, the clustering model relied solely on inflammatory indices. These missing determinants may attenuate phenotype-outcome associations and limit the direct clinical interpretability of these clusters, potentially restricting their application to hypothesis generation. Additionally, other inflammatory biomarkers, such as CRP, ferritin, and ESR, were sparsely measured and therefore excluded. PCA and clustering were performed on a complete case data set without imputation, which may introduce selection bias toward patients with more complete laboratory testing.

Despite these caveats, the study highlights the biological heterogeneity of cardiogenic shock and reinforces the central role of systemic inflammation and immune dysregulation in its pathophysiology. The lack of a simple correspondence between inflammatory phenotype and short-term mortality should not be interpreted as evidence that inflammation is irrelevant in cardiogenic shock; rather, it suggests that inflammation operates within a complex network of hemodynamic, metabolic, and treatment-related factors. In addition, missing values limited serial measurements. Future work integrating high-dimensional immune profiling, hemodynamics, and longitudinal sampling, ideally across multiple cohorts, will be essential to move from descriptive phenotyping toward actionable, mechanism-informed stratification of cardiogenic shock.

## 5. Conclusions

In this multicenter cohort of ICU patients with cardiogenic shock, unsupervised clustering of routinely available hematologic inflammation indices identified two biologically distinct inflammatory phenotypes, including a hyperinflammatory, lymphopenic profile associated with more pronounced organ dysfunction at presentation. Despite clear biological separation, short-term outcomes were similar between phenotypes, suggesting that admission inflammation indices alone may be insufficient for risk stratification at this late stage of illness. Future studies integrating inflammatory profiles with hemodynamics, shock etiology, imaging, and longitudinal immune trajectories, and validating these phenotypes in external cohorts, are needed to determine whether biologically defined subgroups can inform phenotype-guided management strategies.

## Figures and Tables

**Table 1:** Pattern Matrix with Component loading.

	Pattern Matrix Component 1	Pattern Matrix Component 2	Communality
SII	0.99	−0.12	0.90
NLR	0.94	0.01	0.89
AISI	0.64	0.40	0.80
PLR	0.63	0.13	0.49
NPAR	0.60	−0.05	0.33
MLR	−0.09	0.99	0.91
SIRI	0.44	0.67	0.90

Factor loadings for each inflammatory index on the two retained components are shown in Table 1, illustrating that most indices loaded strongly on the first component, whereas MLR and SIRI contributed more to the second. Systemic Inflammatory Index (SII), Neutrophil Lymphocyte Ratio (NLR), Platelet Lymphocyte Ratio (PLR), Monocyte Lymphocyte Ratio (MLR), Neutrophil Percentage-to-Albumin Ratio (NPAR), Systemic Inflammatory Response Index (SIRI), and Aggregate Index of Systemic Inflammation (AISI) contributed to the first component.

**Table 2:** Clinical and Laboratory Values Stratified by Cluster.

Variable	Cluster 1 (N = 52)	Cluster 2 (N = 367)	*p*
Age (years)	72 (63, 83)	67 (57, 77)	0.01
BMI (kg/m^2^)	28.4 (23.8, 31.6)	27.4 (24.0, 32.6)	0.99
ALT (U/L)	68 (31, 792)	100 (43, 410)	0.56
AST (U/L)	206 (52, 1124)	183 (48, 662)	0.65
BUN (mg/dL)	49 (33, 63)	33 (21, 51)	<0.001
Lactic acid (mmol/L)	2.2 (1.2, 4.5)	3.5 (1.8, 7.0)	0.03
INR	1.9 (1.4, 2.5)	1.6 (1.2, 2.7)	0.10
Serum Creatinine (mg/dL)	2.4 (1.3, 3.3)	1.6 (1.1, 2.5)	0.024
SOFA score	10 (7, 11)	10 (6, 13)	0.6
Heart rate (bpm)	115 (101, 135)	115 (98, 130)	0.31
MAP (mm Hg)	53 (47, 58)	55 (43, 64)	0.42
Temperature in Fahrenheit	97.3 (96.8, 97.7)	97.0 (95.4, 97.7)	0.02
Chloride (mmol/L)	95 (90, 101.8)	102 (97, 105)	0.01
Serum Bicarbonate (mmol/L)	19.0 (15.6, 23.4)	18.5 (14.6, 22.1)	0.23
Potassium (mmol/L)	4.6 (4.0, 5.3)	4.3 (3.8, 4.8)	0.05
Sodium (mmol/L)	134 (129, 138)	137 (134, 140)	<0.001
Neutrophils (×10^3^ cells/μL)	9.8 (6.6, 18.0)	7.5 (5.2, 11.0)	<0.001
Platelet (×10^3^ cells/μL)	213 (156, 334)	211 (158, 258)	0.25
Monocytes (×10^3^ cells/μL)	9 (6, 12)	7 (5, 9)	0.002
Lymphocytes (×10^3^ cells/μL)	0.51 (0.38, 0.77)	1.57 (0.97, 2.80)	<0.001
Albumin (g/dL)	3.1 (2.5, 3.4)	3.4 (2.9, 3.8)	0.003
Hemoglobin (g/dL)	11.5 (10.1, 13)	12.4 (10.5, 14.3)	0.01
SII	4869 (3141, 7018)	980 (433, 1796)	<0.001
AISI (×10^9^/L)	3.7 (2.5, 5.9)	6.8 (2.8, 12.0)	<0.001
NLR	20.6 (15, 30)	4.8 (2.3, 9.0)	<0.001
PLR	460.9 (315.4, 601.6)	129.1 (71.2, 201.0)	<0.001
MLR	16.3 (10.4, 23.4)	4.4 (2.2, 7.2)	<0.001
SIRI	164.0 (122.8, 243.1)	33.4 (14.1, 60.0)	<0.001
NPAR	0.27 (0.23, 0.34)	0.22 (0.16, 0.25)	<0.001
Lactate/Albumin	0.77 (0.45, 1.42)	1.03 (0.54, 2.27)	0.09
Total Bilirubin (mg/dL)	1.50 (0.80, 2.50)	0.95 (0.60, 1.60)	0.03

Abbreviations: Body Mass Index (BMI), Alanine Transaminase (ALT), Aspartate Transaminase (AST), Blood Urea Nitrogen (BUN), International Normalized Ratio (INR), Systemic Inflammatory Index (SII), Neutrophil Lymphocyte Ratio (NLR), Platelet Lymphocyte Ratio (PLR), Monocyte Lymphocyte Ratio (MLR), Neutrophil Percentage-to-Albumin Ratio (NPAR), Systemic Inflammatory Response Index (SIRI), and Aggregate Index of Systemic Inflammation (AISI). SOFA: sequential organ failure assesment; MAP: mean arterial pressure.

**Table 3:** Demographics, clinical comorbidities, and outcomes of patients by cluster.

Variables	Overall N = 419	Cluster 1 (N = 52)	Cluster 2 (N = 367)	Odds Ratio for Clusters (OR)	95% Confidence Interval for OR		*p*
					Upper	Lower	
AKI	273 (65.2%)	35 (67.3%)	238 (64.9%)	0.90	0.48	1.67	0.73
PTCA	39 (9.3%)	1 (1.9%)	38 (10.4%)	5.89	0.79	43.84	0.05
Acute MI	131 (31.3%)	13 (25.0%)	118 (32.2%)	1.42	0.73	2.76	0.29
Ethnicity							
Caucasian	321 (76.6%)	39 (75%)	282 (76.8%)				0.20
African American	43 (10.3%)	7 (13.5%)	36 (9.8%)				
Asian	11 (2.6%)	3 (5.8%)	8 (2.2%)				
Hispanic	22 (5.3%)	3 (5.8%)	19 (5.2%)				
Other/Native American	22 (5.3%)	0 (0%)	22 (6.0%)				
Male Gender	252 (60.1%)	39 (75%)	213 (58%)	0.46	0.24	0.89	0.02
Caucasian vs. others	321 (76.6%)	39 (75%)	282 (76.8%)	1.11	0.56	2.12	0.77
Diabetes Mellitus	13 (3.1%)	2 (3.8%)	11 (3.0%)	0.78	0.17	3.59	0.74
Cardiomyopathy	11 (2.6%)	3 (5.8%)	8 (2.2%)	0.37	0.09	1.42	0.13
Cerebrovascular Disease	7 (1.7%)	2 (3.8%)	5 (1.4%)	0.35	0.07	1.83	0.065
CKD	91 (21.7%)	20 (38.5%)	71 (19.4%)	0.39	0.21	0.71	0.002
COPD	38 (9.1%)	7 (13%)	31 (8.5%)	0.60	0.25	1.43	0.24
Hyperlipidemia	22 (5.3%)	2 (3.8%)	20 (5.5%)	1.45	0.33	6.37	0.63
IABP	59 (14.1%)	5 (9.6%)	54 (14.7%)	1.62	0.62	4.26	0.32
ACEi	47 (11.2%)	4 (7.7%)	43 (11.7%)	1.60	0.55	4.65	0.39
ARB	13 (3.1%)	0 (0%)	13 (3.6%)				0.17
Diuretics	121 (28.9%)	13 (25%)	108 (29.5%)	1.26	0.65	2.45	0.50
Emergency CABG	3 (0.7%)	0 (0%)	3 (0.8%)				0.59
Mechanical Ventilation	282 (67.3%)	27 (51.9%)	255 (69.7%)	2.13	1.18	3.83	0.01
Malignancy	13 (3.1%)	4 (7.7%)	9 (2.5%)	0.30	0.09	1.02	0.04
AKI requiring RRT	56 (13.4%)	7 (13.5%)	49 (13.4%)	0.99	0.42	2.32	0.98
Vasopressor use	340 (81.1%)	40 (76.9%)	300 (81.7%)	1.34	0.67	2.70	0.41
Hospital Mortality	146 (34.8%)	21 (40.4%)	125 (34.1%)	0.76	0.42	1.38	0.37
Death within 24 h of admission	78 (18.7%)	7 (13.5%)	71 (19.4%)	2.45	0.51	11.84	0.30
Hospital Length of Stay (Days)	4.1 (2.2, 7.4)	4.0 (1.8, 10.2)	4.1 (2.2, 7.3)				0.86

Abbreviations: Acute Kidney Injury (AKI), Hemodialysis (HD), Percutaneous Coronary Angiography (PTCA), Myocardial Infarction (MI), Chronic Kidney Disease (CKD), Chronic Obstructive Pulmonary Disease (COPD), Intra-Aortic Balloon Pump (IABP), Angiotensin-Converting Enzyme Inhibitor (ACEi), Angiotensin Receptor Blocker (ARB), Coronary Artery Bypass Graft (CABG), Renal Replacement Therapy (RRT).

## Data Availability

The original data presented in the study are openly available in eICU Collaborative Research Database at https://doi.org/10.13026/C2WM1R (accessed on 1 January 2025).

## Supplementary Materials

The following supporting information can be downloaded at: https://www.mdpi.com/article/10.3390/jpm16020096/s1, Table S1: Spearman’s Correlation Matrix of Inflammatory markers of Inflammatory Variables Employed in PCA. Table S2: Pattern Matrix, Commonalities and factor variance of two factor PCA solution. Table S3: Summary of K means cluster analysis. Table S4: Sensitivity Analysis of Clusters Based on Limited PCA Employing Leukocyte Derived Inflammatory Indices. Figure S1: Dendogram using Average Linkage (between groups).
