# Supplementary material for: Unsupervised Clustering of Routine Inflammatory Markers in Cardiogenic Shock Reveals Phenotypic Heterogeneity Without Prognostic Utility
Source: J Pers Med. 2026 Feb 6;16(2):96. doi: 10.3390/jpm16020096 (PMC12941803; doi:10.3390/jpm16020096)
Supplement: Supplementary file 1 [file jpm-16-00096-s001.zip › jpm-4049120-supplementary.pdf]

Supplementary Material

Figure S1 Dendogram using Average Linkage (between groups)

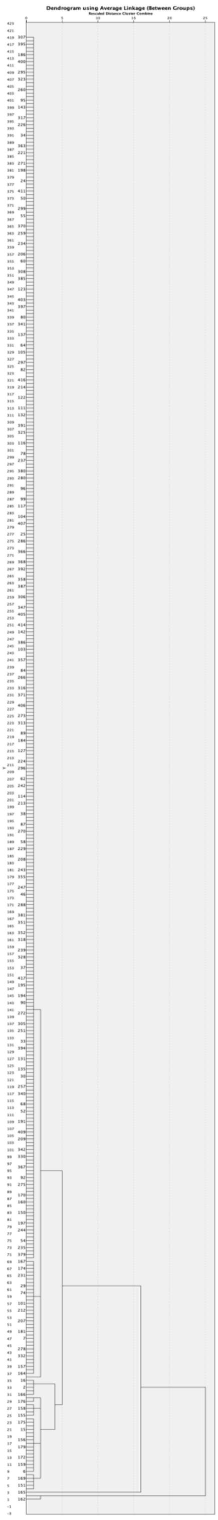

Supplemental Table S1. Spearman's Correlation Matrix of Inflammatory markers of Inflammatory Variables Employed in PCA

		SII	AISI	NLR	PLR	MLR	SIRI	NPAR
**SII**	*Correlation Coefficient*	1.00	0.86	0.91	0.87	0.49	0.77	0.66
	Sig. (2-tailed)		<0.001	<0.001	<0.001	<0.001	<0.001	<0.001
	N	419	419	419	419	419	419	419
**AISI**	*Correlation Coefficient*	0.86	1.00	0.78	0.80	0.69	0.91	0.53
	Sig. (2-tailed)	<0.001		<0.001	<0.001	<0.001	<0.001	<0.001
	N	419	419	419	419	419	419	419
**NLR**	*Correlation Coefficient*	0.91	0.78	1.00	0.80	0.61	0.85	0.70
	Sig. (2-tailed)	<0.001	<0.001		<0.001	<0.001	<0.001	<0.001
	N	419	419	419	419	419	419	419
**PLR**	*Correlation Coefficient*	0.87	0.80	0.80	1.00	0.69	0.72	0.57
	Sig. (2-tailed)	<0.001	<0.001	<0.001		<0.001	<0.001	<0.001
	N	419	419	419	419	419	419	419
**MLR**	*Correlation Coefficient*	0.49	0.69	0.61	0.70	1.00	0.83	0.38
	Sig. (2-tailed)	<0.001	<0.001	<0.001	<0.001		<0.001	<0.001
	N	419	419	419	419	419	419	419
**SIRI**	*Correlation Coefficient*	0.77	0.91	0.85	0.72	0.83	2.00	0.57
	Sig. (2-tailed)	<0.001	<0.001	<0.001	<0.001	<0.001		<0.001
	N	419	419	419	419	419	419	419
**NPAR**	*Correlation Coefficient*	0.66	0.53	0.70	0.57	0.38	0.57	1.00
	Sig. (2-tailed)	<0.001	<0.001	<0.001	<0.001	<0.001	<0.001	
	N	419	419	419	419	419	419	419

Supplemental Table S2. Pattern Matrix, Commonalities and factor variance of two factor PCA solution

**Variable**	**Pattern Matrix Factor 1**	**Pattern Matrix Factor 2**	**Communality**
NPAR	-0.08	0.97	0.89
PLR	0.82	0.08	0.73
NLR	0.52	0.55	0.75
MLR	0.84	-0.12	0.65
**Factor Variance**			
Factor 1 (% Variance)		55.04	
Factor 2 (% Variance)		20.55	
Total (% Variance)		75.59	

Legend: Neutrophil Lymphocyte Ratio (NLR), Platelet Lymphocyte Ratio (PLR), Monocyte Lymphocyte Ratio (MLR), Neutrophil Percentage-to-Albumin Ratio (NPAR)

Supplemental Table S3. Summary of K means cluster analysis

	**Final Cluster 1 Center**	**Final Cluster 2 Center**	**F**	**Effect Size (Cluster 2 – Cluster 1)**	**ANOVA Significance**
**Regression factor score 1**	-0.36	1.16	301	1.52	<0.001
**Regression factor score 2**	-0.33	1.05	221	1.38	<0.001

Legend: F-static (F), Analysis of Variance (ANOVA)

Supplemental Table S4. Sensitivity Analysis of Clusters Based on Limited PCA Employing Leukocyte Derived Inflammatory Indices

Variable	Cluster 1 (N-320)	Cluster 2 (N=99)	Odds Ratio	95% Confidence Interval		p value
				Lower	Upper	
Hemodialysis	36 (12%)	20 (20%)	2.00	1.09	3.60	0.03
Hospital Mortality	106 (33%)	40 (40%)	1.40	0.86	2.10	0.18
Vasopressor use	261 (82%)	79 (79%)	0.89	0.50	1.57	0.69
Need for Mechanical Ventilation	225 (70%)	57 (58%)	0.56	0.36	0.09	0.02
AKI	202 (63%)	71 (72%)	1.48	0.90	2.40	0.12
